# Lifetime exposure to shift work and risk of multiple sclerosis: retrospective and prospective cohort studies in UK Biobank

**DOI:** 10.1136/oemed-2025-110454

**Published:** 2026-07-10

**Authors:** Cathy Wyse, Amber Roguski, Rebecca Maguire, Andrew Coogan, Daniel J Smith, Lorna M Lopez

**Affiliations:** 1 Department of Biology, Maynooth University, Maynooth, Ireland; 2 Centre for Clinical Brain Science, University of Edinburgh, Edinburgh, UK; 3 Department of Psychology, Maynooth University, Maynooth, Ireland

**Keywords:** Shift Work Schedule, Autoimmune Diseases, Circadian Rhythm

## Abstract

**Background:**

Multiple sclerosis (MS) is an autoimmune disease with complex aetiology involving genetic, environmental and lifestyle factors. Shift work disrupts circadian rhythms and is a potential occupational risk factor for MS, with exposure before age 20 thought to be of additional risk.

**Objective:**

To investigate associations between retrospective lifetime shift work exposure and MS diagnosis and between prospective shift work exposure and incident MS over 13 years of follow-up in the UK Biobank cohort.

**Methods:**

Participants that were in work and free of MS at baseline were followed up for 13 years, and lifetime exposure to night, day and mixed shift work was assessed in a subset of participants. Logistic regression and Cox proportional hazard models were applied to test associations between shift work and MS diagnosis, controlling for demographic and lifestyle confounders.

**Results:**

Lifetime exposure to mixed, day or night shift work was not associated with an increased risk of MS diagnosis. No significant associations were observed for early-life shift work exposure or in prospective analysis of those reporting shift work at baseline. Factors such as female sex, smoking, childhood obesity and reduced sunlight exposure were consistently associated with higher MS risk.

**Conclusions:**

There was no evidence for an association between shift work and the risk of MS diagnosis in this cohort, in retrospective analysis of lifetime exposure or in prospective studies of shift work at baseline following 13 years of follow-up. Further research is needed to elucidate mechanisms and latitudinal variations in shift work-related MS risk.

**WHAT IS ALREADY KNOWN ON THIS TOPIC**

- Multiple sclerosis (MS) shows strong geographical and seasonal patterning, implicating photoperiodic and circadian mechanisms as primary pathophysiological factors. Shift work affects both circadian rhythms and daylight exposure and previous studies, largely from high-latitude Scandinavian populations, reported increased MS risk in shift workers and particularly those that carried out shift work in early life.

**WHAT THIS STUDY ADDS**

- MS diagnosis was not associated with lifetime, early-life or cumulative exposure to shift work in this UK population-based cohort suggesting that previously reported associations may not generalise across populations.

**HOW THIS STUDY MIGHT AFFECT RESEARCH, PRACTICE OR POLICY**

- The findings of this study highlight the challenges of accurately quantifying shift work exposure at scale and the potential implications of geographical and photoperiodic contexts. Future research should focus on developing and applying methods for quantifying exposure to shift work at different life stages.

## Introduction

 Multiple sclerosis (MS) is a demyelinating autoimmune disease associated with compromised cognitive, motor and sensory function that affects over 1.8 million people worldwide.^1^ The aetiology of MS is complex and involves genetic (eg, HLA genes) and environmental risk factors (eg, smoking, reduced exposure to daylight, childhood obesity, sleep disruption and viral infection, particularly Epstein-Barr virus).^2–6^ The epidemiology of MS is inexplicably linked to geographical location; its prevalence is rare in tropical regions and more common in areas farther from the Equator in both hemispheres,^7 8^ and the average age of disease onset occurs almost 2 years earlier at high latitudes.^9^ The variable annual day length and dark winters of northern latitudes might mediate this effect, and in support of this, photoperiodic mechanisms are involved in other aspects of the epidemiology of MS. For example, the risk of diagnosis is associated with low exposure to daylight and birth in spring/summer months^8^ and symptoms of MS are more commonly reported in summer.^10–13^ The intensity and timing of exposure to the photoperiod is determined by (1) season, (2) latitude and (3) daylight exposure, all factors associated with MS implicating photoreceptive mechanisms such as circadian timing, seasonality and vitamin D signalling in the pathophysiology of this disease.

Circadian rhythms are endogenously generated oscillations in physiological and behavioural parameters that are entrained to an approximate 24-hour period principally by regular input from the prevalent light-dark cycle. Many aspects of immune function are directly regulated by the circadian clock including immune cell trafficking, cytokine release and response to infection^14^ and circulating immune cells show diurnal variation in humans.^15^ In context of the pathophysiology of MS, genetic disruption of circadian timing mechanisms by lesion of the core clock gene, *Bmal1* increased proinflammatory polarisation of T-cells in an animal model of MS^16^ and impaired myelination during neurodevelopment^17^ providing a mechanistic rationale for how circadian misalignment could both affect MS risk, and increase susceptibility to this in early life. Furthermore, previous work has linked photoperiodic regulation of melatonin with immune function and seasonal patterns of remission and relapse in MS.^11^ The mechanisms that underlie regulation of immune function by the circadian clock and light exposure are key to advancing understanding of how, or if, shift work might increase risk of autoimmune disorders, including MS.

Shift work has previously been associated with increased risk for immune and inflammatory diseases,^18^ effects thought to be mediated in part by its disruptive effects on circadian rhythms. In addition to persistent disruption of circadian rhythms, shift work might also affect the risk of immune disease through reduced opportunities for exposure to daylight, a risk factor with an established role in the pathophysiology of MS. There is evidence that shift work is associated with increased risk of later life MS diagnosis particularly when this occurs before age 20,^19–21^ although this is based exclusively on several studies carried out at high latitudes (Sweden, Denmark). In contrast, there was no overall association between shift work and MS risk in a UK cohort (Nurse’s Health Study) although there was weak evidence for an effect of more than 20 years of exposure.^22^ To our knowledge, the relationship between early life shift work and MS diagnosis has only been reported in Scandinavian cohorts. The UK Biobank contains cross-sectional and lifetime data on exposure to shift work and in this study we leverage these data to investigate the association between shift work and the risk of MS in UK residents.

## Methods

### Participants

The study sample were participants of UK Biobank, a general population cohort study that recruited more than half a million UK residents aged between 37 and 73 years, between 2006 and 2010.^23^ Participants with MS (n=2365) were identified by International Classification of Diseases (ICD)-coded diagnoses (ICD-10-G35; ICD-9-3409), self-reported MS diagnosis, a General Practitioner (GP)-coded diagnosis or through death registration (Data-Field 131043) as previously described^24^ (online supplemental table S1). The age of each participant with MS at the time of diagnosis was derived from the date of the first recorded MS diagnostic code (Data-Field 131044). Participants that were diagnosed with MS before the age of 20 were excluded as were participants that were not in paid or unpaid employment at the time of baseline assessment. Details of all outcome, exposure and covariables are given in online supplemental table S2.

In 2015 a subset of the UK Biobank participants (n=1 20 239) completed an online follow-up questionnaire on their lifetime work history (online supplemental table S3, figure S1 and S2). All participants that completed the online follow-up questionnaire were in paid or unpaid employment.

The prospective study reported here includes the full UK Biobank cohort that self-reported paid or unpaid employment at the time of baseline assessment (n=288 299). The follow-up period was up to 2023, approximately 13 years, depending on the time of the assessment centre appointment of each participant. This group reported their current shift work status at baseline, but no information about the length or type of exposure.

### Measures

Information on participant demography was collected at baseline including age, sex and ethnicity (six-level categorical variable; white, black, mixed, Chinese, Asian, Other). Area-based socio-economic status was assessed using the Townsend score which is derived from census data on housing, employment, social class and car availability. Self-reported sleep duration (hours), chronotype, smoking status, childhood obesity and sunlight exposure and alcohol intake were assessed at baseline (online supplemental table S2 for details). Body mass index (BMI) was measured by trained UK Biobank staff using standardised methods and instruments and WHO criteria used to classify BMI as underweight <18.5, normal weight 18.5–24.9, overweight 25.0–29.9 and obese ≥30.0 kg/m^2.^ Lifestyle variables including smoking, alcohol consumption, time spent outdoors, sleep duration and chronotype were included as potential confounders in the models; although some may also act as mediators, their role as such was not assessed in this study. Further details of the derivation of shift work exposure variables and the covariables included in the model are given in the supplementary data (online supplemental table S2 and S14).

### Data analysis

In our studies of lifetime shift work and MS risk, we considered the observation period from the age of first job until 2023. Participants diagnosed with MS aged less than 20 years were excluded. Multivariable adjusted logistic regression was used to estimate the probability (OR and 95% CI) of diagnosis of MS across participants that had ever worked day, mix and night shifts and their number of hours of exposure across their lifetime job history. The rationale for this approach to the data analysis is outlined in the online supplemental material. The specific impact of early life shift work was tested by including a binary variable of shift work before age 20 as a main effect in all models and by testing interaction with the hours of total exposure to shift work. As illustrated in the directed acyclic graph (online supplemental figure S3), sleep duration and daylight exposure may lie on the causal pathway between shift work and MS. These variables were included as potential confounders and we acknowledge that adjusting for them could have attenuated the estimated effect of shift work. Therefore, sensitivity analyses excluding these potential mediators were performed to assess the robustness of our findings.

We next performed a prospective study of the probability of a future MS diagnosis in the entire UK Biobank cohort that were working at the time of the baseline assessment centre visit. The follow-up period was up to 2023, approximately 13 years, depending on the time of the assessment centre appointment of each participant. Cox proportional hazard models were applied to assess the prospective risk of MS diagnosis in those that reported working shifts compared with non-shift workers. Potential confounders in the prospective study included as covariables were age; sex; ethnicity; Townsend area-deprivation score; physical activity; alcohol intake and smoking status; sleep duration; chronotype; time outdoors. The a priori hypothesis of our prospective and lifetime exposure studies was that shift work affects the probability of an MS diagnosis. All analyses were performed using R V.4.3.2.

## Results

Lifetime work histories taken from the online work questionnaire were available for the subsample of 120 239 of the UK Biobank participants, and there were 575 prevalent cases of MS among them. There were 1098 prevalent cases of MS in the full UK Biobank cohort excluding those participants who were not in work at the time of baseline assessment. The baseline characteristics of the UK Biobank participants who completed the online work history questionnaire, stratified by MS diagnosis are shown in table 1 and in the entire cohort of working participants in table 2. Univariable analysis of sample descriptive statistics showed that participants with MS were more likely to be female, to report childhood obesity, to be smokers currently and to spend less time outdoors (table 1).

**Table 1:** Demographic and lifestyle details of the subset of UK Biobank participants that completed an online work history questionnaire stratified by multiple sclerosis diagnosis

	Control (n=119 665)	Multiple sclerosis (n=574)	P value
Sex			**<0.001**
Female	66 393 (55)	435 (76)	
Male	53 272 (45)	139 (24)	
Age			
Mean (95% CI)	56.07 (56.03 to 56.12)	54.23 (53.63 to 54.83)	**<0.001**
Townsend Index*			
Mean (95% CI)	−1.82 (−1.84 to −1.8)	−1.78 (−2.01 to −1.56)	0.753
Missing	132 (0.1)	1 (0.2)	
BMI category†			**0.004**
Normal weight	44 469 (37)	226 (39)	
Obese	24 069 (20)	118 (21)	
Overweight	50 219 (42)	203 (35)	
Underweight	678 (1)	8 (1)	
Missing	230 (0.2)	19 (3.3)	
Chronotype			0.256
Evening	10 097 (8)	59 (10)	
More evening than morning	29 480 (25)	138 (24)	
More morning than evening	40 423 (34)	201 (35)	
Morning	26 581 (22)	114 (20)	
Missing	13 084 (10.9)	62 (10.8)	
Childhood obesity			**<0.001**
About average	60 667 (51)	298 (52)	
Plumper	19 087 (16)	122 (21)	
Thinner	38 472 (32)	147 (26)	
Missing	1439 (1.2)	7 (1.2)	
Alcohol intake			**<0.001**
Never	6335 (5)	45 (8)	
Special occasions only	10 653 (9)	77 (13)	
One to three times a month	12 972 (11)	69 (12)	
Once or two times a week	29 696 (25)	146 (25)	
Three or four times a week	31 769 (27)	115 (20)	
Daily or almost daily	28 169 (24)	121 (21)	
Missing	71 (0.1)	1 (0.2)	
Time outdoors summer			**<0.001**
Mean hours/day (95% CI)	3.38 (3.37 to 3.39)	2.87 (2.7 to 3.04)	
Missing	5157 (4.3)	24 (4.2)	
Smokers			**<0.001**
Current	7905 (7)	71 (12)	
Never	69 922 (58)	308 (54)	
Previous	41 598 (35)	187 (33)	
Missing	600 (0.5)	3 (0.5)	
Age started smoking			0.483
Mean (95% CI)	17.6 (17.6 to 17.6)	17.4 (16.9 to 17.8)	
Missing	84 881 (70.9)	370 (64.5)	

Data are mean (SD), median (IQR) or percentages.

Bold values indicate statistical significance.

* Negative values indicate relative affluence.

† WHO criteria used to classify BMI as underweight <18.5, normal weight 18.5–24.9, overweight 25.0–29.9 and obese ≥30.0 kg/m2 for both men and women.

BMI, body mass index.

**Table 2:** Demographic, lifestyle and self-reported shift work status at the baseline assessment centre appointment of working participants in the entire UK Biobank cohort stratified by MS diagnosis

	Control (n=287 957)	MS diagnosis (n=342)	P value
Sex			**<0.001**
Female	149 856 (52)	237 (69)	
Male	138 101 (48)	105 (31)	
Age			**<0.001**
Mean (95% CI)	52.8 (52.77 to 52.82)	50.34 (49.65 to 51.03)	
Townsend Index*			0.743
Mean (95% CI)	−1.33 (−1.34 to −1.32)	−1.27 (−1.60 to −1.95)	
Missing	403 (0.1)	0 (0)	
BMI category			0.801
Normal weight	94 731 (33)	118 (35)	
Obese	68 515 (24)	75 (22)	
Overweight	122 243 (42)	146 (43)	
Underweight	1355 (0)	1 (0)	
Missing	1113 (0.4)	2 (0.6)	
Chronotype			0.411
Evening	23 878 (8)	34 (10)	
More evening than morning	74 836 (26)	96 (28)	
More morning than evening	90 633 (31)	97 (28)	
Morning	66 783 (23)	85 (25)	
Missing	31 827 (11.1)	30 (8.8)	
Sleep duration category			**0.133**
Long sleep (<9 hours)	12 891 (4)	22 (6)	
Optimum sleep (7–9 hours)	200 126 (70)	238 (70)	
Short sleep (<7 hours)	73 984 (26)	78 (23)	
Missing	956 (0.3)	4 (1.2)	
Childhood obesity			0.025
About average	142 839 (50)	185 (54)	
Plumper	46 701 (16)	63 (18)	
Thinner	93 916 (33)	88 (26)	
Missing	4501 (1.6)	6 (1.8)	
Time outdoors summer			**0.010**
Mean (95% CI)	3.46 (3.45 to 3.46)	3.11 (2.85 to 3.77)	
Missing	17 607 (6.1)	28 (8.2)	
Smokers			**<0.001**
Current	30 801 (11)	63 (18)	
Never	164 867 (57)	183 (54)	
Previous	91 513 (32)	94 (27)	
Missing	776 (0.3)	2 (0.6)	
Current shift worker			0.175
Always	22 024 (8)	31 (9)	
Never/rarely	235 704 (82)	277 (81)	
Sometimes	21 167 (7)	18 (5)	
Usually	6071 (2)	11 (3)	
Missing	2991 (1.0)	5 (1.5)	
Current night shift worker			0.232
Always	7101 (2)	12 (4)	
Never/rarely	24 400 (8)	27 (8)	
Sometimes	14 200 (5)	14 (4)	
Usually	3924 (1)	11 (3)	
Missing	2991 (1.0)	5 (1.5)	

Data are mean (SD), median (IQR) or percentages.

Bold values indicate statistical significance.

* Negative values indicate relative affluence.

BMI, body mass index; MS, multiple sclerosis.

Descriptive statistics and univariable analysis showed multiple unfavourable health and lifestyle related parameters in shift workers in cross-sectional analysis of those reporting shift work at baseline assessment (online supplemental table S6), and in longitudinal studies of lifetime histories (online supplemental table S4). Further information on the characteristics of the participants according to their early-life shift work exposure are available in online supplemental table S5.

**Figure 1:**
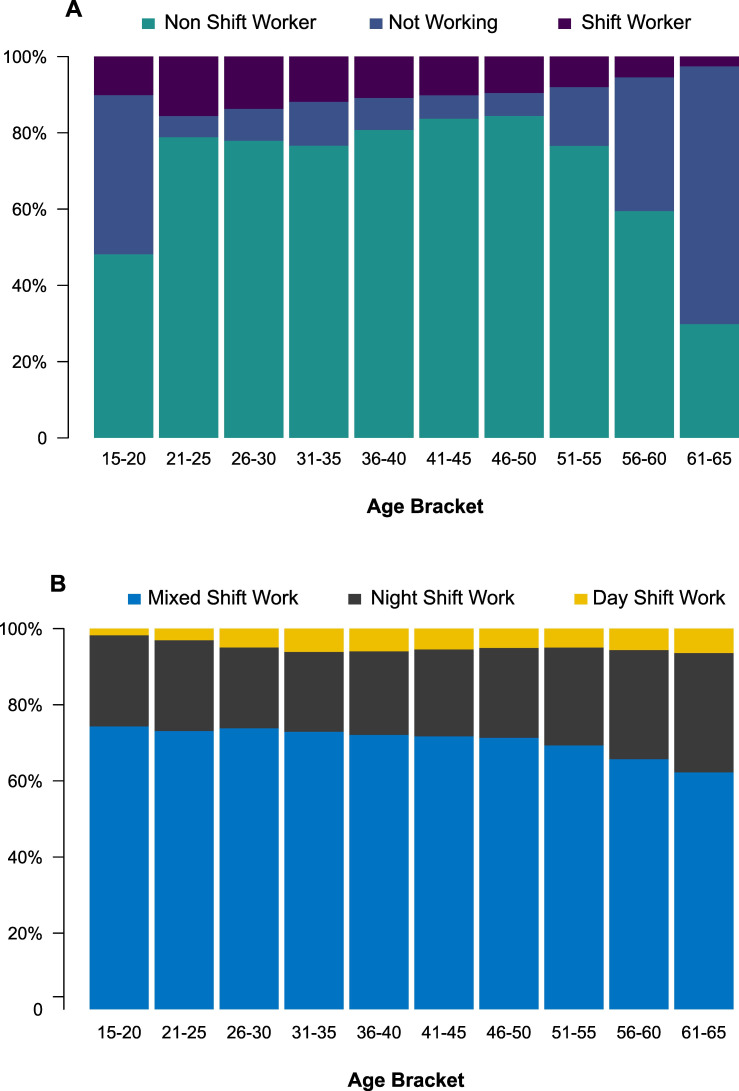
Proportion of shiftworkers at each age bracket (A) and proportion of shiftwork type at each age bracket (B).

Proportionally more participants reported retrospectively that they undertook shift work in younger 21–25 age groups compared with other age groups, with the proportion reporting shift work at older age groups decreasing thereafter (figure 1). Considering only shift workers, the proportion of night shift workers increased in the older age categories, with the largest proportion of night shift workers among the 61–65 age categories. (figure 1)

### Lifetime shift work exposure and risk of MS diagnosis

In participants who reported lifetime exposure to shift work, logistic regression models showed that there was no association between being a shift worker at any time in life and subsequent diagnosis of MS (online supplemental table S8). When the absolute level of exposure to shift work was quantified in hours over the lifetime, there was no effect on the probability of MS diagnosis (online supplemental table S9, table 3). There was no effect of reporting shift work in early life (before the age of 20 years), as assessed by a main effect and by interaction with the total hours of shift work (online supplemental tables S9–S11). When the type of shift work exposure was investigated separately (day, mix and night shift work), there was no significant association between MS risk and exposure to mixed, day or night shift work. (online supplemental table S21). It should be noted that the mixed shift work category had an OR and 95% CI (1.12 to 1.01–1.24, p=0.094) that was most close to reaching statistical significance.

**Table 3:** Details of lifetime shift work history stratified by multiple sclerosis diagnosis in the subset of UK Biobank participants that completed the online lifetime work history questionnaire

	Control (n=1 19 665)	Multiple sclerosis (n=574)	P value
Lifetime day shift work exposure			0.848
Mean hours (95% CI)	23 162 (22 629 to 23 695)	22 418 (15 591 to 29 245)	
Lifetime night shift work exposure			0.371
Mean hours (95% CI)	19 301 (18 271 to 20 331)	12 677 (4242 to 21 112)	
Lifetime mixed shift work exposure			0.935
Mean hours (95% CI)	39 680 (39 212 to 40 147)	39 960 (32 969 to 46 950)	
Most common occupation of shift workers			0.139
Administration	1564 (6%)	7 (6%)	
Associate professional	10 393 (39%)	65 (51%)	
Elemental	1804 (7%)	4 (3%)	
Machine operating	2457 (9%)	11 (9%)	
Managers	2400 (9%)	10 (8%)	
Professional	2901 (11%)	16 (13%)	
Sales	699 (3%)	2 (2%)	
Service	2635 (10%)	11 (9%)	
Trades	1863 (7%)	3 (2%)	
Early life night shift worker	9491 (8%)	53 (9%)	0.283
Early life shift worker	12 095 (10%)	65 (11%)	0.371
Ever a shift worker	26 641 (22%)	127 (22%)	0.877
Ever a night shift worker	20 410 (17%)	96 (17%)	0.977

These findings were replicated in a sensitivity analysis using a data subset that included only shift workers, indicating their robustness to the impact of zero inflation of the outcome variable, MS diagnosis (online supplemental tables S18–S20). Across all of the models, smoking, childhood obesity, time spent outdoors in summer and female sex were associated with an increased probability of MS diagnosis (online supplemental tables S8–S10). Although the case numbers are small, 574 participants with MS and 119 665 controls (with 22% of participants classified as shift workers, the study was powered to detect effects previously reported (OR ≳1.5). The numbers of MS diagnoses in those reporting shift work in early life are even smaller, 65 with MS compared with 12 160 without, consequently, this study had moderate power (~55–60%) to detect previously reported effect sizes.

To assess the possibility that exposure to outdoor light or sleep duration might act as a mediator as well as a potential confounder of any relationship between shift work and MS diagnosis, we performed a sensitivity analysis by running the models with this variable excluded which did not change the results, indicating that in this case, outdoor light exposure and sleep had no mediatory effect and could be considered as a covariable (online supplemental tables S15–S17).

### Shift work at baseline and prospective risk of MS diagnosis

**Figure 2:**
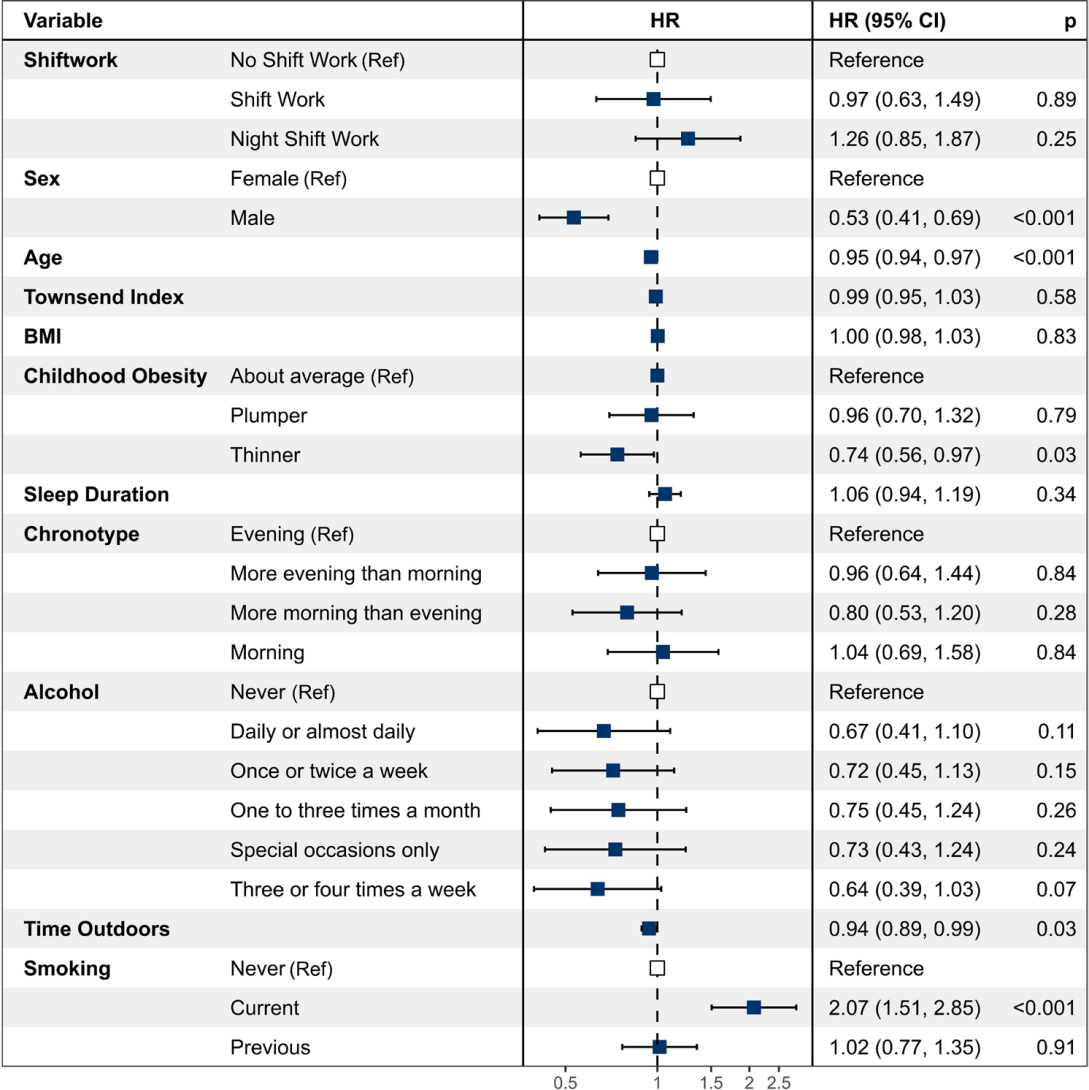
Forest plot showing hazard ratios and 95% CI for the risk of MS diagnosis after approximately 12 years of follow up. BMI, body mass index.

In our prospective studies, Cox regression analysis showed there was no increased risk of MS diagnosis during follow-up in those UK Biobank participants who reported shift work or night shift work at baseline compared with those working participants who did not do shift work (figure 2). There were significant differences between the shift workers and non-shift workers across all lifestyle and health-related parameters at baseline (online supplemental table S7). In agreement with our findings in the lifetime shift work studies, sex (female), lower exposure to outdoor light, increased childhood obesity and smoking were associated with increased risk of MS diagnosis but there was no association with shift work or night shift work. The prospective cohort of 285 303 individuals free of MS diagnosis at baseline and 342 incident cases over 10 years had adequate statistical power to detect moderate associations between shift work and MS risk (HRs ≳1.5) as previously reported.^19 21 22^

## Discussion

This study of working UK Biobank participants showed that neither lifetime exposure to mixed (rotating) day or night shift work was associated with an increased risk of MS diagnosis. There was no evidence of increased risk of diagnosis of MS in participants who reported shift work in early life (<20 years) or who reported having ever been a shift worker at any point of their lifetime job history. The absolute dose of exposure to all shift work was quantified in terms of hours of lifetime exposure and had no relationship with MS diagnosis. Prospective studies of those participants who reported shift work at a single time point (baseline assessment) did not identify any change in the risk of MS diagnosis over approximately 13 years of follow-up compared with non-shift workers. These findings in UK Biobank are in contrast to previous studies that associated exposure to shift work with an increased risk of MS, particularly after early life exposure.^20 21^ However, it must be acknowledged that the long latency of MS means that baseline assessment of shift work used in this prospective study may not fully capture exposures during the etiologically relevant windows earlier in life. Consequently, our prospective analyses may underestimate associations if critical exposures occurred before the baseline assessment.

The UK Biobank job-history questionnaire collected detailed information on the lifetime exposure to shift work, but there was considerable heterogeneity between participants in terms of the duration, type and proportion of each job that were shift work jobs making calculation of exposure difficult. There was no information on the frequency or direction of shift rotation and circadian misalignment, consequently, an important biological mechanism through which the adverse effects of shift work might be mediated was not completely captured. Nevertheless, the main finding of our study was that there was no association between shift work and MS diagnosis, regardless of the underlying mechanism. Previous research has often used total years of shift work, or the number of night shifts per week to represent shift work exposure. In the case of the UK Biobank, lifetime shift work data, years of exposure to shift work does not account for variable proportions of shift work between jobs or that the number of night shifts per week was highly variable between and within jobs. None of these parameters account for total work hours, which could vary between shift and non-shift workers. For these reasons, and in contrast to many previous studies, we elected to quantify lifetime exposure to shift work in hours as well as to adjust for total hours of work. The quantification of exposure to shift work is further complicated by the variability in shift duration and frequency, rotation patterns and the proportion of night work in late/night shifts. These difficulties in defining and quantifying shift work are major obstacles to investigating its impact on health, and could account for the discrepancies between our findings in UK Biobank and previous studies of shift work and MS risk.^19–21^

A significant finding of this study was a failure to reproduce the effect of early life shift work on MS risk reported in previous studies,^19–21^ which might be explained by the comparatively small number of MS cases in UK Biobank, inconsistency in metrics to quantify shift work or by the diverse factors that might determine exposure to early life shift work between countries.

The adverse effects of shift work could be biased by social and demographic differences between shift workers in different occupations or countries, and it is of concern that most studies reporting adverse effects of shift work were based in healthcare settings. It is a strength of our studies that UK Biobank shift workers represented diverse occupations, nevertheless, socio-economic and cultural differences between early life shift workers in Scandinavia and the UK could account for our failure to reproduce association between early life shift work and MS diagnosis in UK Biobank. Future studies should address the social and cultural factors which affect the likelihood of shift work, and that could contribute to its adverse effects particularly in relation to the effects of early life shift work. It should also be considered that exposure to the lifestyle factors associated with shift work (smoking, alcohol, sedentary behaviour, sleep deprivation) early in life could establish poor health behaviours that persist into adulthood with negative consequences on lifetime health outcomes. An effect of shift work before age 20 years on lifetime MS risk is not excluded by our findings and warrants further investigation. Our studies corroborate previous studies in UK Biobank and others that identify childhood obesity, decreased exposure to daylight and smoking as independent risk factors for MS.^5 24^ Sleep deprivation was proposed as a mediator of associations between early life shift work and MS^25 26^ but there was no association between sleep duration at baseline and MS risk in our prospective studies. There was no evidence to support a mediating role for sleep or outdoor light exposure on the relationship between shift work and MS diagnosis but the question of causality was beyond the scope of the present study. Prospective research is required to elucidate the mechanisms that drive the long-term adverse effects of shift work.

Studies in animal models have provided biologically plausible links between circadian disruption and immune dysregulation that could put shift workers at increased risk of developing autoimmune disorders. For example, genetic disruption of the core components of the molecular clock (BMAL1) in myeloid cells exacerbated autoimmune neuroinflammation and altered monocyte and T-cell activity relevant to central nervous system autoimmunity.^16^ Furthermore, studies in humans have shown that simulated night shift schedules lead to phase shifts and desynchronisation of cytokine secretion by monocytes and T lymphocytes.^27^ Nevertheless, epidemiological evidence remains uncertain which probably reflects the difficulties of quantifying exposure to shift work, and the influence of the multiple confounding factors that comprise this complex social and biological construct.

Low vitamin D is associated with MS and shift work but its direct pathophysiological role is unproven.^28 29^ This uncertainty, in addition to the multiple effects of light on health that are not captured by vitamin D, led us to elect to use a behavioural proxy measure, ‘time outdoors in summer’, to indicate lifetime exposure to daylight. Nevertheless, vitamin D synthesis could be compromised by lack of outdoor light exposure due to shift work and further research tracking vitamin D and its relationship with immune function in shift workers is warranted.

The strength of this study is the large sample size and detailed demographic and lifestyle information that was available in UK Biobank. It is a strength of this study that the shift workers were drawn from a diverse range of occupations, and that detailed information on the timing and intensity of shift work across their lifetime was available.

Nevertheless, the results of this study must be considered in the context of several significant limitations. MS diagnosis was based on healthcare records and the numbers of cases were low and small effects might have escaped detection, however, we estimate that the analysis had statistical power to detect associations between shift work and MS diagnosis previously described. The UK Biobank is compromised by a significant lack of ethnic diversity and a healthy volunteer bias that precludes generalisation of our findings. The self-reported nature of the data is subject to recall bias and classification error and cannot be used to determine the causal direction of relationships between shift work and health outcomes. Prospective studies are required to investigate the long-term implications of shift work carried out by adolescents and young adults.

The data on shift work collected at baseline provide no information on the intensity or duration of exposure. Early life work history is most likely subject to recall bias, and there was no information available on early life disease, sleep or other health-related parameters. Exposures strongly associated with MS such as infectious mononucleosis status were not available for the whole UK Biobank cohort and could not be included in the analyses. Self-selection into and out of shift work is likely to bias all investigations of its health impacts, especially for permanent nightshift.^30^ Finally, shift work is a complex exposure with consequences for many aspects of health and lifestyle and which involves diverse and overlapping mechanisms such as night-at-light exposure, short and poor quality sleep, substance use and poorer nutritional behaviours, which may also act as predictors of health outcomes independently of shift work.^31^ Consequently, there is a high risk of residual confounding in this study and in all investigations of the health outcomes of shift work.

The associations between early life shift work and MS risk reported at high latitudes were not reproduced in these studies in UK Biobank. The disruptive effect of shift work on circadian and seasonal biology, vitamin D production and other photoreceptive mechanisms may be augmented at high latitudes and the comparative risks of shift work in different regions of the world warrant further investigation. Despite our findings of no association between exposure to shift work and the risk of MS in UK Biobank, the previous associations reported in Scandinavian cohorts^19–21^ and a small effect of long-term exposure in the UK Nurses Health study^22^ justify further investigation on how shift work affects immune function and monitoring of its association with the prevalence of MS and other autoimmune disorders.

## Supplementary material

10.1136/oemed-2025-110454 online supplemental file 1

10.1136/oemed-2025-110454 online supplemental file 2

## Data Availability

Data may be obtained from a third party and are not publicly available.
