# Supplementary material for: Lifetime exposure to shift work and risk of multiple sclerosis: retrospective and prospective cohort studies in UK Biobank
Source: Occup Environ Med. 2026 Jul 10;83(4):e110454. doi: 10.1136/oemed-2025-110454 (PMC13479283; doi:10.1136/oemed-2025-110454)
Supplement: Supplementary file 1 [file oemed-83-4-s001.pdf]

# Derivation of UK Biobank Lifetime Shiftwork Metrics

## Introduction

Data are taken from UKB Category 130

Details of lifetime employment history collected at online follow-up. Participants were asked to enumerate their work history. Periods of 6 months or longer during which they were in paid full time employment for at least 15 hours each week have been labelled “jobs”, with all other periods labelled “gaps”.

Data are processed to give parameters used to compare the effects of shiftwork at different ages. There are sometimes more than one job per year of age, and these could be shiftwork or not, and could be different occupations.

https://biobank.ndph.ox.ac.uk/showcase/label.cgi?id=130 (https://biobank.ndph.ox.ac.uk/showcase/label.cgi?id=130)

R code used to derive lifetime shiftwork metrics is here (Projects/Lifetime-Shiftwork/extract_SW_metrics191223.R) Two for loops iterate over each job for each eid to make a table that summarises job history for each person. These tables were stored in a folder of csv files. Because some people had two jobs per year, the data are expressed as hours per year. No person had more than 39 jobs in their lifetime. The life_jobtable csv is a list of all jobs history for all eids and another loop fast-read these files and summarised type of shiftwork, occupation, hours per year and dose exposure to nightshift at each age bracket. The age brackets were: 15-20, 21-25, 26-30, 31-35, 36-40, 41-45, 46-50, 51-55, 56-60, 61-65.

# 1. Occupations

Occupation coding of job type 4-digit SOC2000 coding UKB df22617. This coding was used to create a factor variable. Because some people had more than one job at each year, the final variable (per age bracket) table stores job occupation as a vector

Major groups used to make a factor variable (job_occupation)

| Level | Label | Description |
|---|---|---|
| 1 | Managers | Managers and Senior Officials |
| 2 | Prof | Professional Occupations |
| 3 | Assoc_prof | Associate Professional and Technical Occupations |
| 4 | Admin | Administrative and Secretarial Occupations |
| 5 | Trades | Skilled Trades Occupations |
| 6 | Service | Personal Service Occupations |
| 7 | Sales | Sales and Customer Service Occupations |
| 8 | Machine | Process, Plant and Machine Operatives |
| 9 | Element | Elementary Occupations |

# 2. Shiftwork Duration

Some people had more than one job per year, so not possible to assume a shiftwork job was totally shiftwork, although all jobs were more than 15 hours per week. For this reason, the number of hours per job was calculated and used to express job duration as hours per year. This was calculated using UKB df22605 (number of hours per week, numeric) but in the case of this being a missing value, hours per week was inputted from UKB df22604 (hours per week, categorical) assuming the midpoint of each category

| Level | Code | Label |
|---|---|---|
| 1 | -1520 | 15 to less-than-20 hours |
| 2 | -2030 | 20 to less-than-30 hours |
| 3 | -3040 | 30 to 40 hours |
| 4 | -4000 | Over 40 hours |

When the categorical value for work hours was also missing, the data for hours per week was inputted as 35 hours.

# 3. Work Type

Work type was categorised as non-shiftwork, day shift, night shift and mix shift in a variable called “SW_type”. If this variable has a missing value, the person was taking a gap year from work or did not enter data. Either way, a missing value is taken as not working. This variable records whether a person did shiftwork during this job, but not the amount. For example, someone might do shiftwork only for the first year of a ten year job. The proportion of shiftwork was assessed using UKB df 22651.0, 22641.0, 22631.0.

Participants were first asked: “Which shift pattern(s) did you follow for this job?” Participants who indicated they worked a mixture of day and night shifts were then asked: “Did you work a mixture of day and night shifts for the whole of this job?” Day-shifts were defined as work in nomal daytime hours or morning, afternoon or evening work. Night-shifts were defined as work for at least 3 hours between midnight and 5am. Mixed was both. These were categorical variable for all three shiftwork types (day, night, mix)

| Level | Coding | Label |
|---|---|---|
| 1 | 0 | Shift pattern was worked for some (but not all) of job |
| 2 | 1 | Shift pattern was worked for whole of job |
| 3 | 9 | This type of shift pattern was not worked during job |

To get the percent shiftwork per year for this job, the years of shiftwork was divided by total years in the job. 1 means the whole job was spent in shiftwork or 0.5 half of the years. Difficult to account for this because the age that shiftwork was done is critical for this study. Best compromise, was to re-code a job with less than 40% SW as not SW and more than 40% as SW. This was inputted into the SW_type variable so there are only shiftworkers and non shiftworkers.

# 4. Shiftwork Severity

The number of hours of night shiftwork was taken to represent the severity of shiftwork. There were questions asked about the number of night shifts and the length of each shift so it was possible to calculate the "dose" of night shiftwork per year for each type (mix and night).

The final shiftwork variables were

| Variable name | Description |
|---|---|
| agebracket | the 5-year window |
| bracket_total_hr | total hours worked per year |
| bracket_total_hr_daySW | total hours day shift worked per year |
| bracket_total_hr_nightSW | total hours night shift worked per year |
| bracket_total_hr_mixSW | total hours mix shift worked per year |
| bracket_SW_occup | occupation while doing shiftwork (they could have had different occupations for non-shiftwork jobs in the same bracket) |
| bracket_SW_per_work | proportion of work years that was any kind of shiftwork - this will normalise to the hours of work |
| bracket_nightSW_per_work | proportion of work years that was night shiftwork - this will normalise to the hours of work |
| bracket_daySW_per_work | proportion of work years that was day shiftwork - this will normalise to the hours of work |
| bracket_mixSW_per_work | proportion of work years that was mix shiftwork - this will normalise to the hours of work |

# 5. Participants that Developed MS

The participants with a diagnosis of MS are taken from UKB variables 131042 and 131043

| Data Field 131042 | Date G35 first reported (multiple sclerosis) |
|---|---|
| 1900-01-01 | Code has no event date |
| 1901-01-01 | Code has event date before participant's date of birth |
| 1902-02-02 | Code has event date matching participant's date of birth |
| 1903-03-03 | Code has event date after participant's date of birth and falls in the same calendar year as date of birth |
| 1909-09-09 | Code has event date in the future and is presumed to be a place-holder or other system default |
| 2037-07-07 | Code has event date in the future and is presumed to be a place-holder or other system default |

| Data Field 131043 | Source of report of G35 (multiple sclerosis) |
|---|---|
| 20 | Death register only |

| Data Field 131043 | Source of report of G35 (multiple sclerosis) |
|---|---|
| 21 | Death register and other source(s) |
| 30 | Primary care only |
| 31 | Primary care and other source(s) |
| 40 | Hospital admissions data only |
| 41 | Hospital admissions data and other source(s) |
| 50 | Self-report only |
| 51 | Self-report and other source(s) |

Participants that had a diagnosis before age 20 were excluded. Unknown or uncertain diagnosis dates are not excluded, but it is not possible to know if they were diagnosed before 20
