# Supplementary material for: Lifetime exposure to shift work and risk of multiple sclerosis: retrospective and prospective cohort studies in UK Biobank
Source: Occup Environ Med. 2026 Jul 10;83(4):e110454. doi: 10.1136/oemed-2025-110454 (PMC13479283; doi:10.1136/oemed-2025-110454)
Supplement: Supplementary file 2 [file oemed-83-4-s002.DOC]

**Supplementary Methods**

*Rationale for the Data Analysis Approach*

We elected to use logistic regression models to investigate general associations between shift work and MS diagnosis due to heterogeneity in the timing and nature of exposure and diagnosis in the lifetime job history dataset. In Model 1, we applied logistic regression to model the probability of MS diagnosis in those that had ever done any shift work (since most shift workers reported a combination of day, mix and night shift work). In Model 2, we addressed the possibility of a dose-response by modelling the risk of MS as a function of quantitative exposure to shift work (total lifetime hours of day, mixed, and night shift work), where most participants had zero hours exposure. Day, night and mixed shift work present different challenges and in Model 3 we addressed specific effects of exposure to each type of shift work on MS risk. To account for these multiple comparisons across the shift work variables, we applied the Benjamini-Hochberg procedure to control the False Discovery Rate (FDR). The Benjamini–Hochberg false discovery rate correction was applied to account for multiple testing while controlling the expected proportion of false positives, without assuming independence between outcomes. This approach preserves statistical power when examining multiple related measures of shift work. To assess the robustness of this approach, we repeated the analysis in Models 1-3 in a sensitivity analysis that included only participants who undertook shift work. The purpose of this sensitivity analysis was to assess potential bias due to the large number of zero in the MS risk outcome variable.

Table S1	Source of MS diagnosis (UK Biobank data field source of first G35 report).

| **Source of MS report** | **n** |
| --- | --- |
| Death register only | 2 |
| Death register and other source(s) | 100 |
| Primary care only | 272 |
| Primary care and other source(s) | 437 |
| Hospital admissions data only | 137 |
| Hospital admissions data and other source(s) | 1118 |
| Self-report only | 299 |

Table S2	Details of calculation of shift work exposure and covariables

| **Field ID** | **Field Name** | **Sub-Category** |
| --- | --- | --- |
| 31 | Sex | Baseline characteristics |
| 21003 | Age at assessment centre | Reception |
| 21000 | Ethnic background | Ethnicity |
| 54 | UK Biobank assessment centre | Reception |
| 6138 | Qualifications | Education |
| 20119 | Current employment status – corrected | Employment |
| 3426 | Job involves night shift work | Employment |
| 826 | Job involves shift work | Employment |
| 20115 | Country of Birth (non-UK origin) | Early life factors |
| 1647 | Country of birth (UK/elsewhere) | Early life factors |
| 1737 | Sunburn as a child | Early life factors |
| 20116 | Smoking status | Smoking |
| 1558 | Alcohol intake frequency. | Alcohol |
| 1160 | Sleep duration | Lifestyle and environment |
| 1180 | Morning/evening person (chronotype) | Lifestyle and environment |
| 90012 | Overall acceleration average | Acceleration averages |
| 1001 | Body mass index (BMI) | Body size measures |
| 22200 | Year of birth | Work environment |
| 22601 | Job coding | Work environment |
| 22617 | Job code - historical | Work environment |
| 22602 | Year job started | Work environment |
| 22603 | Year job ended | Work environment |
| 22620 | Job involved shift work | Work environment |
| 22630 | Day shifts worked | Work environment |
| 22631 | Period spent working day shifts | Work environment |
| 1001 | Body mass index (BMI) | Body size measures |
| 22200 | Year of birth | Work environment |
| 22601 | Job coding | Work environment |
| 22617 | Job code - historical | Work environment |
| 22602 | Year job started | Work environment |
| 22603 | Year job ended | Work environment |
| 22620 | Job involved shift work | Work environment |
| 22630 | Day shifts worked | Work environment |
| 22631 | Period spent working day shifts | Work environment |
| 22640 | Mixture of day and night shifts worked | Work environment |
| 22641 | Period spent working mix of day and night shifts | Work environment |
| 22642 | Usual length of each night shift during mixed shift periods | Work environment |
| 22643 | Number of night shifts worked monthly during mixed shift periods | Work environment |
| 22650 | Night shifts worked | Work environment |
| 22641 | Period spent working mix of day and night shifts | Work environment |
| 22642 | Usual length of each night shift during mixed shift periods | Work environment |
| 22643 | Number of night shifts worked monthly during mixed shift periods | Work environment |
| 22650 | Night shifts worked | Work environment |
| 22651 | Period spent working night shifts | Work environment |
| 22643 | Number of night shifts worked monthly during mixed shift periods | Work environment |
| 22650 | Night shifts worked | Work environment |
| 22651 | Period spent working night shifts | Work environment |
| 22652 | Usual length of each night shift during night shift periods | Work environment |
| 22653 | Number of night shifts worked monthly during night shift periods | Work environment |
| 22651 | Period spent working night shifts | Work environment |
| 22652 | Usual length of each night shift during night shift periods | Work environment |
|  |  |  |

**Covariables**

Baseline information was collected at an assessment centre using a questionnaire and information was collected on the demographic status of the participants included age at baseline, sex (male/female), ethnicity (White, Black, Mixed, Chinese, Asian, Other). Area-based socioeconomic status derived from postcode of residence, using the Townsend score which is derived from census data on housing, employment, social class and car availability.

Sleep duration was derived from the touchscreen question "*About how many hours’ sleep do you get in every 24 hours?"*. Habitual sleep duration was self-reported in hours per 24h. Participants were categorised as morning or evening chronotype by the touchscreen question, *“Do you consider yourself to be, Definitely a 'morning' person (coded 1), more a 'morning' than 'evening' person (coded 2), more an 'evening' than a 'morning' person (coded 3), definitely an 'evening' person (coded 4).”* Participants that elected not to answer or that answered ‘Don’t know” were set to missing.

Smoking status was assessed by self-report of having never smoked (coded 0, never), an ex-smoker (coded 1, former), smokes occasionally (coded 3, current), smokes on most or all days (coded 3, current). The age of starting smoking was included as a nested variable with the smoking status variable.

The frequency of alcohol intake was categorised as daily or almost daily, 3-4 times/week, 1-2 times/week, 1-3 times/month, special occasions only or never. The frequency and volume of alcohol intake were self-reported.

Body mass index (BMI was measured by trained UK Biobank staff using standardized methods and instruments.35 and WHO criteria used to classify BMI as underweight <18.5, normal weight 18.5-24.9, overweight 25.0-29.9 and obese ≥30.0 kg/m2.

Childhood obesity was assessed using self-reported variable, *"When you were 10 years old, compared to average would you describe yourself as: Thinner, Plumper, About average*” with options “*Do not know*” or “*Prefer not to answer*” set to missing.

Sunlight exposure in youth was estimated using a self-reported variable on sunburn in childhood. Participants were asked “*Before the age of 15, how many times did you suffer sunburn that was painful for at least 2 days or caused blistering?"* (UKB DF1737).

Lifestyle variables including smoking, alcohol consumption, time spent outdoors, sleep duration, and chronotype were included as potential confounders in the models; although some may also act as mediators, their role as such was not assessed in this study.

**Shift Work Exposure**

UK Biobank collected self-reported data on shift work status at baseline (cross-sectional) in the entire cohort, and on lifetime exposure to shift work in a subset of participants (longitudinal). Shift work exposure was assessed using questionnaires on two occasions: (i) participants were asked about current shift work at their baseline assessment on entry to UK Biobank and (ii) a subset of participants completed follow up online assessment of lifetime exposure to shift work. The content of the questionnaires is shown in the Supplementary Information (Tables S13-14) as well as a screenshot of the panel of questions presented to participants (Figure S1-2).

At the initial assessment (2006–2010) participants who reported they were employed were asked about current shift work using a touchscreen question. The definition shown to participants was: Shift work is “a work schedule that falls outside of the normal daytime working hours of 9 am to 5 pm; this may involve working afternoons, evenings or nights or rotating through these kinds of shifts.” Participants were asked: “Does your work involve shift work?” (Field 826), with response options “Never/rarely, Sometimes, Usually, Always, Do not know, Prefer not to answer.” Participants who reported any shift work were next asked whether their work involved night shifts — defined as “a work schedule that involves working through the normal sleeping hours, for instance working through the hours from 12 am to 6 am.” These variables were used to define current shift workers (any shift work), current night shift workers and current non-shift workers in the full cohort (Supplementary Data (Tables S13-14, Figures S1-2).

A subset of participants completed an online follow-up Employment History questionnaire (Category 130; job-level employment history), and the variable (Field 22620) “Job involved shift work”) captured whether shift work was part of each reported job (binary variable, “Yes”, “No”). For each job, participants were asked: "Did you ever work shifts (day and/or night shifts) for this job?" where shift work referred to work schedules that fall outside the normal daytime working hours (9 am – 5 pm), including afternoon, evening, night, or mixed shifts. Participants who reported shift work were next asked whether each of their jobs involved night, day or mixed shifts. Night shifts were defined as work for at least 3 hours between midnight and 5am, Day shifts were work in normal daytime hours or morning afternoon or evening shifts and Mixed shifts were both Day and Night shifts. For each of Day, Night and Mixed shift work participants were asked whether they worked that type “….for the whole of this job?" The response options were “Shift pattern was worked for some (but not all) of job”, “	Shift pattern was worked for whole of job” and “Shift pattern was not worked during job”. This categorisation allowed for the fact that shift work usually involves proportions of day and night shifts. However, the rotation speed or regularity was not captured by the UK Biobank shift work questionnaire. The online work history questionnaire also collected data on the lifetime number of jobs and the type of occupations, and length and frequency of Mixed and Night shifts for each job.

These data were used to derive the following binary variables: ever a shift worker, ever a night shift worker and shift work before age 20 years old. The frequency and length of night shifts for each job were used to derive variables to assess the burden of shift work exposure. Exposure to shift work was quantified in hours in order to accommodate jobs that did not entail 100% shift work and variable weekly work hours. The frequency and duration of night, day and mixed shifts for each job were used to derive variables assessing the burden of shift work exposure. To account for jobs that did not involve 100% shift work and variable weekly and daily work hours, exposure to shift work was quantified in hours. These variables were subsequently standardised (z-score) to improve interpretability of the effect of shift work and to mitigate issues related to the small unit size.

*Classification of Occupation*

Occupation was categorised according to the Standard Occupational Classification (SOC2010, Office for National Statistics, UK Government)^1^ during a verbal interview with UK Biobank personnel and based on the skill level allocated to standard occupations; Categories 1-3 (professional and managerial), and 4-9 (trade, administrative and services) were merged, to create a binary variable that described professional (coded 1) and non-professional occupations (administrative, trade and services) (coded 0) (Supplementary Data 2).

^1^De Matteis S, *at al* Occupational self-coding and automatic recording (OSCAR): a novel web-based tool to collect and code lifetime job histories in large population-based studies. Scand J Work Environ Health. 2017 Mar 1;43(2):181-186. doi: 10.5271/sjweh.3613).

Table S3	Demographic, lifestyle and work details of the subset of UK Biobank participants that completed the lifetime work history questionnaire stratified by ever having done any kind of shift work.

|  | **Ever a Shift Worker** | | |
| --- | --- | --- | --- |
|  | *No (N=93471)* | *Yes (N=26768)* | *p* |
| **Sex** |  |  | **< 0.001** |
| Female | 53544 (57 %) | 13284 (50 %) |  |
| Male | 39927 (43 %) | 13484 (50 %) |  |
| **Age** |  |  | **< 0.001** |
| mean (95% CI) | 56.34 (56.29, 56.39) | 55.11 (55.01, 55.2) |  |
| **Townsend Index*** |  |  | **< 0.001** |
| mean (95% CI) | -1.91 (-1.93, -1.89) | -1.5 (-1.53, -1.46) |  |
| **BMI Category** |  |  | **< 0.001** |
| Normal weight | 36371 (39 %) | 8324 (31 %) |  |
| Obese | 17309 (19 %) | 6878 (26 %) |  |
| Overweight | 39021 (42 %) | 11401 (43 %) |  |
| Underweight | 579 (1 %) | 107 (0 %) |  |
| **Chronotype** |  |  | **< 0.001** |
| Evening | 7652 (8 %) | 2504 (9 %) |  |
| More evening than morning | 22807 (24 %) | 6811 (25 %) |  |
| More morning than evening | 32044 (34 %) | 8580 (32 %) |  |
| Morning | 20595 (22 %) | 6100 (23 %) |  |
| **Child Obesity** |  |  | 0.125 |
| About average | 47512 (51 %) | 13453 (50 %) |  |
| Plumper | 14844 (16 %) | 4365 (16 %) |  |
| Thinner | 29964 (32 %) | 8655 (32 %) |  |
| **Alcohol Intake** |  |  | **< 0.001** |
| Daily or almost daily | 22736 (24 %) | 5554 (21 %) |  |
| Never | 4809 (5 %) | 1571 (6 %) |  |
| Once or twice a week | 23001 (25 %) | 6841 (26 %) |  |
| One to three times a month | 9783 (10 %) | 3258 (12 %) |  |
| Special occasions only | 7978 (9 %) | 2752 (10 %) |  |
| Three or four times a week | 25110 (27 %) | 6774 (25 %) |  |
| **Time Outdoors Summer** |  |  | **< 0.001** |
| mean (95% CI) | 3.28 (3.27, 3.29) | 3.71 (3.68, 3.74) |  |
| **Maternal Smoker** |  |  | **< 0.001** |
| Do not know | 10301 (11 %) | 3061 (11 %) |  |
| Yes | 22518 (24 %) | 7450 (28 %) |  |
| **Sunburn as Child** |  |  | **< 0.001** |
| Do not know | 22575 (24 %) | 6133 (23 %) |  |
| more than 5 times | 5919 (6 %) | 1924 (7 %) |  |
| none | 33981 (36 %) | 9662 (36 %) |  |
| once or twice | 20376 (22 %) | 5971 (22 %) |  |
| three to five times | 10064 (11 %) | 2936 (11 %) |  |
| **Smoker** |  |  | **< 0.001** |
| Current | 5619 (6 %) | 2357 (9 %) |  |
| Never | 56490 (60%) | 13740 (51 %) |  |
| Previous | 31189 (33 %) | 10594 (40 %) |  |
| **Age Started Smoking** |  |  | **< 0.001** |
| Mean (95% CI) | 17.44 (17.72, 17.81) | 17.15 (17.08, 17.23) |  |

Data are mean (SD), median (interquartile range), or percentages.

*Negative values indicate relative affluence.

Table S4	Demographic, lifestyle and work details of the subset of UK Biobank participants that completed the lifetime work history questionnaire stratified by ever having done any kind of night shift work.

|  | **Ever a Night Shift Worker** | | |
| --- | --- | --- | --- |
|  | *No (n=99733)* | *Yes (n=20506)* | *p* |
| **Sex** |  |  | **< 0.001** |
| Female | 57616 (58 %) | 9212 (45 %) |  |
| Male | 42117 (42 %) | 11294 (55 %) |  |
| **Age** |  |  | **< 0.001** |
| mean (95% CI) | 56.24 (56.2, 56.29) | 55.18 (55.08, 55.29) |  |
| **Townsend Index** |  |  | **< 0.001** |
| mean (95% CI) | -1.88 (-1.89, -1.86) | -1.54 (-1.58, -1.5) |  |
| **BMI Category** |  |  | **< 0.001** |
| Underweight | 617 (1 %) | 69 (0 %) |  |
| Normal weight | 38595 (39 %) | 6100 (30 %) |  |
| Overweight | 41562 (42 %) | 8860 (43 %) |  |
| Obese | 18754 (19 %) | 5433 (26 %) |  |
| **Chronotype** |  |  | **< 0.001** |
| Evening | 8159 (8 %) | 1997 (10 %) |  |
| More evening than morning | 24390 (24 %) | 5228 (25 %) |  |
| More morning than evening | 34153 (34 %) | 6471 (32 %) |  |
| Morning | 22027 (22 %) | 4668 (23 %) |  |
| **Child Obesity** |  |  | 0.089 |
| Thinner | 31918 (32 %) | 6701 (33 %) |  |
| About average | 50697 (51 %) | 10268 (50 %) |  |
| Plumper | 15895 (16 %) | 3314 (16 %) |  |
| **Alcohol Intake** |  |  | **< 0.001** |
| Never | 5202 (5 %) | 1178 (6 %) |  |
| Special occasions only | 8659 (9 %) | 2071 (10 %) |  |
| One to three times a month | 10586 (11 %) | 2455 (12 %) |  |
| Once or twice a week | 24660 (25 %) | 5182 (25 %) |  |
| Three or four times a week | 26631 (27 %) | 5253 (26 %) |  |
| Daily or almost daily | 23935 (24 %) | 4355 (21 %) |  |
| **Time Outdoors Summer** |  |  | **< 0.001** |
| mean hours (95% CI) | 3.29 (3.27, 3.3) | 3.8 (3.77, 3.84) |  |
| **Maternal Smoke** |  |  | **< 0.001** |
| Do not know | 11020 (11 %) | 2342 (11 %) |  |
| Yes | 24161 (24 %) | 5807 (28 %) |  |
| **Sunburn as a Child** |  |  | **< 0.001** |
| Do not know | 24048 (24 %) | 4660 (23 %) |  |
| None | 36245 (36 %) | 7398 (36 %) |  |
| Once or twice | 21794 (22 %) | 4553 (22 %) |  |
| Three to five times | 10725 (11 %) | 2275 (11 %) |  |
| More than 5 times | 6329 (6 %) | 1514 (7 %) |  |
| **Smoker** |  |  | **< 0.001** |
| Current | 6124 (6 %) | 1852 (9 %) |  |
| Never | 59831 (60 %) | 10399 (51 %) |  |
| Previous | 33588 (34 %) | 8195 (40 %) |  |
| **Age Started Smoking** |  |  | **< 0.001** |
| Mean (95% CI) | 17.73 (17.68, 17.78) | 17.10 (17.02, 17.19) |  |
|  |  |  |  |
| **Early Life Night Shift Work** |  |  | **< 0.001** |
| Yes | 87 (0 %) | 9457 (46 %) |  |
| **Early Life Shift Work** |  |  | **< 0.001** |
| Yes | 2226 (2%) | 9934 (48%) |  |

Data are mean (SD), median (interquartile range), or percentages.

*Negative values indicate relative affluence.

Table S5	Demographic, lifestyle and work details of the subset of UK Biobank participants that completed the lifetime work history questionnaire stratified by having done any kind of shift work aged less than 20 years old (early life shift work).

|  | **Early Life Shift Work** | | |
| --- | --- | --- | --- |
|  | *No (N=108,079)* | *Yes (N=12,160)* | *p* |
| **Sex** |  |  | **< 0.001** |
| Female | 60857 (56 %) | 5971 (49 %) |  |
| Male | 47222 (44 %) | 6189 (51 %) |  |
| **Age** |  |  | **< 0.001** |
| mean (95% CI) | 56 (56, 56) | 55 (55, 55) |  |
| **Townsend*** |  |  | **< 0.001** |
| mean (95% CI) | -1.84 (-1.85, -1.82) | -1.67 (-1.72, -1.62) |  |
| **BMI Category** |  |  | **< 0.001** |
| Normal weight | 41336 (38 %) | 3359 (28 %) |  |
| Obese | 20736 (19 %) | 3451 (28 %) |  |
| Overweight | 45131 (42 %) | 5291 (44 %) |  |
| Underweight | 656 (1 %) | 30 (0 %) |  |
| **Chronotype** |  |  | **< 0.001** |
| Evening | 9139 (8 %) | 1017 (8 %) |  |
| More evening than morning | 26594 (25 %) | 3024 (25 %) |  |
| More morning than evening | 36680 (34 %) | 3944 (32 %) |  |
| Morning | 23716 (22 %) | 2979 (24 %) |  |
| **Child Obesity** |  |  | < 0.85 |
| About average | 54783 (51 %) | 6182 (51 %) |  |
| Plumper | 17231 (16 %) | 1978 (16 %) |  |
| Thinner | 34777 (32 %) | 3842 (32 %) |  |
| **Alcohol Intake** |  |  | **< 0.001** |
| Daily or almost daily | 25839 (24 %) | 2451 (20 %) |  |
| Never | 5675 (5 %) | 705 (6 %) |  |
| Once or twice a week | 26635 (25 %) | 3207 (26 %) |  |
| One to three times a month | 11600 (11 %) | 1441 (12 %) |  |
| Special occasions only | 9413 (9 %) | 1317 (11 %) |  |
| Three or four times a week | 28853 (27 %) | 3031 (25 %) |  |
| **Time Outdoors in Summer** |  |  | **< 0.001** |
| mean hours (95% CI) | 3.31 (3.3, 3.32) | 3.96 (3.91, 4) |  |
| **Maternal Smoking** |  |  | **< 0.001** |
| Do not know | 11909 (11 %) | 1453 (12 %) |  |
| Yes | 26395 (24 %) | 3573 (29 %) |  |
| **Sunburn as a Child** |  |  | 0.043 |
| Do not know | 25818 (24 %) | 2890 (24 %) |  |
| more than 5 times | 7079 (7 %) | 764 (6 %) |  |
| None | 39110 (36 %) | 4533 (37 %) |  |
| once or twice | 23672 (22 %) | 2675 (22 %) |  |
| three to five times | 11762 (11 %) | 1238 (10 %) |  |
| **Smoker** |  |  | **< 0.001** |
| Current | 6124 (6 %) | 1852 (9 %) |  |
| Never | 59831 (60 %) | 10399 (51 %) |  |
| Previous | 33588 (34 %) | 8195 (40 %) |  |
| **Age Started Smoking** |  |  | **< 0.001** |
| mean (95% CI) | 17.72 (17.47, 17.76) | 16.82 (16.72, 16.92) |  |
| **Ever a Shift Worker** | 14641 (14 %) |  |  |
| **Ever a Night Shift Worker** | 10572 (10 %) | 9934 (82 %) |  |

Data are mean (SD), median (interquartile range), or percentages.

*Negative values indicate relative affluence.

Table S6	Demographic and lifestyle details at baseline (assessment centre visit) of all working participants of UK Biobank stratified by current shift work status.

|  | **Shift Work Status at Baseline** | | |
| --- | --- | --- | --- |
|  | *Non Shift Worker (N=235981)* | *Shift Worker (N=49322)* | *p* |
| **Sex** |  |  | **<0.001** |
| Female | 125920 (53 %) | 22147 (45 %) |  |
| Male | 110061 (47 %) | 27175 (55 %) |  |
| **Age** |  |  | **<0.001** |
| mean (95% CI) | 52.95 (52.92, 52.98) | 51.88 (51.81, 51.94) |  |
| **Townsend** |  |  | **<0.001** |
| mean (95% CI) | -1.5 (-1.51, -1.49) | -0.55 (-0.58, -0.52) |  |
| **Ethnicity** |  |  | **<0.001** |
| Asian | 4033 (2 %) | 1753 (4 %) |  |
| Black | 3284 (1 %) | 1922 (4 %) |  |
| Chinese | 794 (0 %) | 237 (0 %) |  |
| Mixed | 1537 (1 %) | 454 (1 %) |  |
| Other | 1631 (1 %) | 972 (2 %) |  |
| White | 224052 (95 %) | 43816 (89 %) |  |
| **BMI Category** |  |  | **<0.001** |
| Normal weight | 80643 (34 %) | 13072 (27 %) |  |
| Obese | 53355 (23 %) | 14568 (30 %) |  |
| Overweight | 100030 (42 %) | 21236 (43 %) |  |
| Underweight | 1133 (0 %) | 192 (0 %) |  |
| **Childhood Obesity** |  |  | **<0.001** |
| About average | 118016 (50 %) | 23544 (48 %) |  |
| Plumper | 37841 (16 %) | 8422 (17 %) |  |
| Thinner | 76832 (33 %) | 16240 (33 %) |  |
| **Maternal Smoking** |  |  | **<0.001** |
| Do not know | 26256 (11 %) | 5811 (12 %) |  |
| No | 144659 (61 %) | 28423 (58 %) |  |
| Yes | 61951 (26 %) | 14268 (29 %) |  |
| **Sunburn as a Child** |  |  | **<0.001** |
| Do not know | 53917 (23 %) | 11099 (23 %) |  |
| more than 5 times | 14385 (6 %) | 2554 (5 %) |  |
| None | 89382 (38 %) | 20216 (41 %) |  |
| once or twice | 51554 (22 %) | 10384 (21 %) |  |
| three to five times | 24811 (11 %) | 4670 (9 %) |  |
| **Chronotype** |  |  | **<0.001** |
| Evening | 18768 (8 %) | 4842 (10 %) |  |
| More evening than morning | 60955 (26 %) | 13284 (27 %) |  |
| More morning than evening | 76247 (32 %) | 13630 (28 %) |  |
| Morning | 54578 (23 %) | 11555 (23 %) |  |
| **Self-Reported Health** |  |  | **<0.001** |
| Excellent | 45950 (19 %) | 6867 (14 %) |  |
| Fair | 42054 (18 %) | 11861 (24 %) |  |
| Good | 142560 (60 %) | 28809 (58 %) |  |
| Poor | 4813 (2 %) | 1525 (3 %) |  |
| **Sleep Duration** |  |  | **<0.001** |
| mean (95% CI) | 7.07 (7.06, 7.07) | 6.93 (6.92, 6.94) |  |
| **Sleep Category** |  |  | **<0.001** |
| Long (< 7 hr) | 10074 (4 %) | 2573 (5 %) |  |
| Optimal (7 – 9 hr) | 168133 (71 %) | 30217 (61 %) |  |
| Short (> 9 hr) | 57199 (24 %) | 16185 (33 %) |  |
| **Alcohol Intake** |  |  | **<0.001** |
| Daily or almost daily | 48338 (20 %) | 7693 (16 %) |  |
| Never | 13248 (6 %) | 4255 (9 %) |  |
| Once or twice a week | 64925 (28 %) | 13795 (28 %) |  |
| One to three times a month | 27188 (12 %) | 6604 (13 %) |  |
| Special occasions only | 22237 (9 %) | 6561 (13 %) |  |
| Three or four times a week | 59928 (25 %) | 10370 (21 %) |  |
| **Time Outdoors in Summer** |  |  | **<0.001** |
| mean (95% CI) | 3.32 (3.31, 3.33) | 4.11 (4.09, 4.14) |  |
| **Smoking Status** |  |  | **<0.001** |
| Current | 22995 (10 %) | 7532 (15 %) |  |
| Never | 137118 (58 %) | 26177 (53 %) |  |
| Previous | 75293 (32 %) | 15433 (31 %) |  |
| **Age Started Smoking** |  |  | **<0.001** |
| mean (95% CI) | 17.42 (17.39, 17.46) | 17.05 (16.98, 17.11) |  |
| **Current Night Shift** |  |  | **<0.001** |
| Always |  | 7093 (14 %) |  |
| Never/rarely |  | 24103 (49 %) |  |
| Sometimes |  | 14140 (29 %) |  |
| Usually |  | 3913 (8 %) |  |

Table S7	Adjusted Cox regression analysis of the association between shift work reported at baseline in all working participants of UK Biobank, categorized as either day shift only or any type of night shift and showing the risk of MS diagnosis over 13 years of follow-up. These data correspond to the forest plot shown in Figure 2.

|  | **MS Diagnosis** | | |
| --- | --- | --- | --- |
|  | *HR* | *95 % CI* | *p* |
| **Shift Work Status** |  |  |  |
| No Shift Work (ref) |  |  |  |
| Day Shift Work | 0.967 | 0.638 – 1.489 | 0.881 |
| Night Shift Work | 1.249 | 0.841 – 1.85 | 0.269 |
| **Sex** |  |  |  |
| Female (ref) |  |  |  |
| Male | 0.532 | 0.140-0.690 | **< 0.001** |
| **Age** | 0.951 | 0.934-0.968 | **< 0.001** |
| **Townsend Index** | 0.988 | 0.950-1.028 | 0.563 |
| **Childhood Obesity** |  |  | **0.031** |
| About average (ref) |  |  |  |
| Plumper | 0.956 | 0.695-1.314 |  |
| Thinner | 0.938 | 0.560-0.972 |  |
| **Sleep Duration Category** |  |  |  |
| Optimal (7-9h) (ref) |  |  |  |
| Short (< 7h) | 0.963 | 0.628-1.448 | 0.793 |
| Long (< 9h) | 1.314 | 0.810-2.134 | 0.268 |
| **Chronotype** |  |  |  |
| Morning (ref) |  |  |  |
| More morning than evening | 0.964 | 0.561-1.040 | 0.087 |
| More evening than morning | 0.920 | 0.677-1.250 | 0.596 |
| Evening | 0.953 | 0.628-1.448 | 0.824 |
| **Alcohol Intake** |  |  |  |
| Never (ref) |  |  |  |
| Special occasions only | 0.731 | 0.430-1.243 | 0.247 |
| 1-3 times a month | 0.749 | 0.449-1.249 | 0.269 |
| 1-2 times a week | 0.720 | 0.454-1.141 | 0.162 |
| 3-4 times a week | 0.6417 | 0.396-1.037 | 0.070 |
| Daily | 0.671 | 0.407-1.107 | 0.118 |
| **Time Outdoors in Summer** | 0.9391 | 0.887-0.993 | **0.029** |
| **Smoking Status** |  |  |  |
| Never (ref) |  |  |  |
| Previous | 1.016 | 0.767-1.347 | **0.907** |
| Current | 2.065 | 1.501-2.840 | **< 0.001** |

Table S8	Model 1: Adjusted logistic regression analysis examining the association between every having done shift work and MS diagnosis, controlling for demographic and lifestyle factors in the subset of UK Biobank participants that completed the online work history questionnaire. This analysis (Model 1) accounts for reporting any type of shiftwork.

|  | **MS Diagnosis** | | |
| --- | --- | --- | --- |
| *Predictors* | *OR* | *CI* | *p* |
| **Ever a Shift Worker (hours)** |  |  |  |
| No (ref) |  |  |  |
| Yes | 0.96 | 0.73 – 1.25 | 0.764 |
| **Early Life Shift Work** |  |  |  |
| No (ref) |  |  |  |
| Yes | 1.26 | 0.88 – 1.80 | 0.205 |
| **Total Work (hours)** | 0.83 | 0.75-0.92 | 0.001 |
| **Sex** |  |  |  |
| Female (ref) |  |  |  |
| Male | 0.51 | 0.41 – 0.63 | <0.001 |
| **Age** | 0.98 | 0.97 – 0.99 | 0.001 |
| **Townsend Index** | 0.98 | 0.95 – 1.01 | 0.136 |
| **Childhood Obesity** |  |  |  |
| About average (ref) |  |  |  |
| Plumper | 1.17 | 0.94 – 1.46 | 0.152 |
| Thinner | 0.81 | 0.66 – 0.99 | 0.038 |
| **Alcohol Intake** |  |  |  |
| Never (ref) |  |  |  |
| Special occasions only | 0.98 | 0.67 – 1.46 | 0.932 |
| 1-3 times a month | 0.79 | 0.53 – 1.18 | 0.233 |
| 1-2 times a week | 0.75 | 0.53 – 1.08 | 0.111 |
| 3-4 times a week | 0.59 | 0.41 – 0.86 | **0.005** |
| Daily | 0.71 | 0.49 – 1.03 | 0.064 |
| **Time Outdoors in Summer** | 0.91 | 0.86 – 0.95 | <0.001 |
| **Smoking Status** |  |  |  |
| Never (ref) |  |  |  |
| Previous | 1.22 | 1.00 – 1.47 | **0.045** |
| Current | 2.43 | 1.84 – 3.17 | **<0.001** |

Table S9	Model 2: Adjusted logistic regression analysis examining the association between total lifetime shift work dose (hours) and MS diagnosis, controlling for demographic and lifestyle factors in the subset of UK Biobank participants that completed the online work history questionnaire. This analysis accounts for total exposure to any type of shift work and tests for interaction with the status of having done shiftwork early in life (< 20 years old).

|  | **MS Diagnosis** | | |
| --- | --- | --- | --- |
| *Predictors* | *OR* | *CI* | *p* |
| **Total Shift Work (hours)** | 1.17 | 0.84 – 1.60 | 0.322 |
| **Early Life Shift Work** |  |  |  |
| No (ref) |  |  |  |
| Yes | 1.08 | 0.73 – 1.55 | 0.677 |
| **Total Shift work * Early Life Shift Work** | 0.96 | 0.76 – 1.19 | 0.695 |
| **Total Work (hours)** | 1.17 | 0.84 – 0.091 | **<0.001** |
| **Sex** |  |  |  |
| Female (ref) |  |  |  |
| Male | 0.51 | 0.41 – 0.63 | **<0.001** |
| **Age** | 0.98 | 0.97 – 0.99 | **0.002** |
| **Townsend Index** | 0.98 | 0.94 – 1.01 | 0.123 |
| **Childhood Obesity** |  |  |  |
| About average (ref) |  |  |  |
| Plumper | 1.17 | 0.94 – 1.46 | 0.151 |
| Thinner | 0.81 | 0.66 – 0.99 | **0.038** |
| **Alcohol Intake** |  |  |  |
| Never (ref) |  |  |  |
| Special occasions only | 0.99 | 0.67 – 1.47 | 0.946 |
| 1-3 times a month | 0.79 | 0.54 – 1.18 | 0.243 |
| 1-2 times a week | 0.76 | 0.53 – 1.09 | 0.123 |
| 3-4 times a week | 0.59 | 0.42 – 0.87 | **0.005** |
| Daily | 0.71 | 0.50 – 1.04 | 0.074 |
| **Time Outdoors in Summer** | 0.90 | 0.86 – 0.95 | **<0.001** |
| **Smoking Status** |  |  |  |
| Never (ref) |  |  |  |
| Previous | 1.22 | 1.00 – 1.47 | **0.047** |
| Current | 2.42 | 1.83 – 3.15 | **<0.001** |

Table S10	Model 3: Adjusted logistic regression analysis examining the association between lifetime shift work dose (hours) for each type of shiftwork (day, night and mix) and MS diagnosis, controlling for demographic and lifestyle factors in the subset of UK Biobank participants that completed the online work history questionnaire. This analysis (Model 3) addresses the specific effects of exposure to day, mixed and night shiftwork.

|  | **MS Diagnosis** | | |
| --- | --- | --- | --- |
| *Predictors* | *OR* | *CI* | *p* |
| **Day Shift Work (hours)** | 1.00 | 0.90 – 1.10 | 0.930 |
| **Mixed Shift Work (hours)** | 1.12 | 1.01 - 1.24 | **0.031** |
| **Night Shift Work (hours)** | 0.94 | 0.83 – 1.06 | 0.595 |
| **Early Life Shift Work** |  |  |  |
| No (ref) |  |  |  |
| Yes | 1.05 | 0.75 – 1.45 | 0.766 |
| **Total Work (hours)** | 0.81 | 0.73 – 0.91 | **<0.001** |
| **Sex** |  |  |  |
| Female (ref) |  |  |  |
| Male | 0.50 | 0.40 – 0.62 | **<0.001** |
| **Age** | 0.98 | 0.97 – 0.99 | **0.002** |
| **Townsend Index** | 0.98 | 0.95 – 1.01 | 0.131 |
| **Childhood Obesity** |  |  |  |
| About average (ref) |  |  |  |
| Plumper | 1.17 | 0.94 – 1.46 | 0.151 |
| Thinner | 0.81 | 0.66 – 0.99 | **0.038** |
| **Alcohol Intake** |  |  |  |
| Never (ref) |  |  |  |
| Special occasions only | 0.99 | 0.67 – 1.47 | 0.951 |
| 1-3 times a month | 0.79 | 0.54 – 1.18 | 0.244 |
| 1-2 times a week | 0.75 | 0.53 – 1.09 | 0.121 |
| 3-4 times a week | 0.59 | 0.41 – 0.86 | **0.005** |
| Daily | 0.71 | 0.50 – 1.04 | 0.071 |
| **Time Outdoors in Summer** | 0.90 | 0.86 – 0.95 | **<0.001** |
| **Smoking Status** |  |  |  |
| Never (ref) |  |  |  |
| Previous | 1.22 | 1.00 – 1.47 | **0.045** |
| Current | 2.43 | 1.84 – 3.16 | **<0.001** |

Table S11	Sensitivity analysis: Adjusted logistic regression analysis examining the association between lifetime shift work dose (hours) for total hours of shift work exposure and MS diagnosis, controlling for demographic and lifestyle factors in the subset of UK Biobank participants that completed the online work history questionnaire. This sensitivity analysis is restricted to those participants that did shiftwork.

|  | **MS Diagnosis** | | |
| --- | --- | --- | --- |
| *Predictors* | *OR* | *CI* | *p* |
| **Total Shift Work (hours)** | 1.61 | 1.03 – 2.45 | **0.032** |
| **Early Life Shift Work** |  |  |  |
| No (ref) |  |  |  |
| Yes | 1.48 | 0.88 – 2.49 | 0.135 |
| **Total Shift Work (hours) * Early Life Shift Work** | 0.82 | 0.63 – 1.08 | 0.149 |
| **Total Work (hours)** | 0.81 | 0.62 – 1.04 | 0.101 |
| **Sex** |  |  |  |
| Female (ref) |  |  |  |
| Male | 0.48 | 0.30 – 0.76 | **0.002** |
| **Age** | 0.98 | 0.95 – 1.00 | 0.057 |
| **Townsend Index** | 0.92 | 0.85 – 0.98 | **0.016** |
| **Childhood Obesity** |  |  |  |
| About average (ref) |  |  |  |
| Plumper | 1.06 | 0.64 – 1.70 | 0.816 |
| Thinner | 0.77 | 0.49 – 1.19 | 0.253 |
| **Alcohol Intake** |  |  |  |
| Never (ref) |  |  |  |
| Special occasions only | 1.13 | 0.53 – 2.63 | 0.759 |
| 1-3 times a month | 0.59 | 0.25 – 1.46 | 0.238 |
| 1-2 times a week | 0.73 | 0.36 – 1.66 | 0.423 |
| 3-4 times a week | 0.55 | 0.26 – 1.28 | 0.140 |
| Daily | 0.69 | 0.32 – 1.61 | 0.361 |
| **Time Outdoors in Summer** | 0.94 | 0.85 – 1.03 | 0.178 |
| **Smoking Status** |  |  |  |
| Never (ref) |  |  |  |
| Previous | 1.29 | 0.84 – 1.99 | 0.241 |
| Current | 3.31 | 1.93 – 5.50 | **<0.001** |

Table S12	Sensitivity analysis: Adjusted logistic regression analysis examining the association between lifetime shift work dose (hours) for total hours of shift work exposure for each type of shift work and MS diagnosis, controlling for demographic and lifestyle factors in the subset of UK Biobank participants that completed the online work history questionnaire. This sensitivity analysis is restricted to those participants that did shiftwork.

|  | **MS Diagnosis** | | |
| --- | --- | --- | --- |
| *Predictors* | *OR* | *CI* | *p* |
| **Day Shift Work (hours)** | 1.04 | 0.92 – 1.15 | 0.500 |
| **Mixed Shift Work (hours)** | 1.19 | 1.03 – 1.37 | **0.021** |
| **Night Shift Work (hours)** | 0.99 | 0.85 – 1.08 | 0.868 |
| **Early Life Shift Work** |  |  |  |
| No (ref) |  |  |  |
| Yes | 1.14 | 0.78 – 1.68 | 0.489 |
| **Total Work (hours)** | 0.81 | 0.62 – 1.04 | 0.106 |
| **Sex** |  |  |  |
| Female (ref) |  |  |  |
| Male | 0.47 | 0.29 – 0.74 | **0.001** |
| **Age** | 0.98 | 0.95 – 1.00 | 0.070 |
| **Townsend Index** | 0.92 | 0.85 – 0.98 | **0.016** |
| **Childhood Obesity** |  |  |  |
| About average (ref) |  |  |  |
| Plumper | 1.06 | 0.64 – 1.69 | 0.828 |
| Thinner | 0.77 | 0.49 – 1.19 | 0.251 |
| **Alcohol Intake** |  |  |  |
| Never (ref) |  |  |  |
| Special occasions only | 1.14 | 0.53 – 2.64 | 0.750 |
| 1-3 times a month | 0.59 | 0.25 – 1.46 | 0.238 |
| 1-2 times a week | 0.73 | 0.36 – 1.66 | 0.424 |
| 3-4 times a week | 0.55 | 0.26 – 1.28 | 0.139 |
| Daily | 0.69 | 0.32 – 1.61 | 0.366 |
| **Time Outdoors in Summer** | 0.94 | 0.85 – 1.02 | 0.162 |
| **Smoking Status** |  |  |  |
| Never (ref) |  |  |  |
| Previous | 1.29 | 0.83 – 1.98 | 0.246 |
| Current | 3.31 | 1.93 – 5.50 | **<0.001** |

Table S13	Work Schedule Questions (Current Shift Work): Touchscreen Questionnaire

| **Data Field** | **Question** | **Response Options** | **Notes** |
| --- | --- | --- | --- |
| 826 | Does your work involve shift work? | 1 = Never/rarely 2 = Sometimes 3 = Usually 4 = Always −1 = Do not know −3 = Prefer not to answer | If more than one current job, answer for **main job only**. Shift work defined as work outside normal daytime hours (9:00–17:00), including afternoon, evening, night, or rotating shifts.  If response = 1 (Never/rarely), go to 3426. |
| 3426 | Does your work involve night shifts? | 1 = Never/rarely 2 = Sometimes 3 = Usually 4 = Always −1 = Do not know −3 = Prefer not to answer | If more than one current job, answer for **main job only**. Night shifts defined as working during normal sleeping hours (e.g. 00:00–06:00). |

**Online Follow-Up Employment Questionnaires**

Participants were sent an invitation by email to complete an online employment questionnaire that aimed to assess longitudinal work and occupational exposure data beyond the data collected at baseline. It captured detailed work history and environment data that can be linked with health outcomes such as respiratory health, chronic disease and ageing.

Participants were asked to provide the follow information about every period of employment of 6 months or longer and at least 15 hours per week during their lifetime:

• Job start and end year

• Standardised job coding

• Work hours (exact and categorical)

• Whether shifts or night shifts were worked

• Workplace environmental exposures (noise, heat, dust, chemicals)

• Health outcomes such as breathing difficulties during work periods

Table S14	UK Biobank Online Follow-up Questionnaire: Work Environment – Employment History Variables

| **Field ID** | **Variable Description** | **Data Type** | **Coding (Values / Meaning)** |
| --- | --- | --- | --- |
| **22599** | Number of jobs held | Integer | Integer count of jobs ≥6 months/≥15 hr/wk |
| **22601** | Job coding (SOC) | Categorical | Standard occupational classification codes |
| **22602** | Year job started | Integer | Four-digit year (YYYY) |
| **22603** | Year job ended | Integer | Four-digit year (YYYY) |
| **22604** | Work hours – lumped category | Categorical | -1520 15 to less-than-20 hours  -2030 20 to less-than-30 hours  -3040 30 to 40 hours  4000 Over 40 hours |
| **22605** | Work hours per week – exact value | Integer | Exact number of hours worked per week |
| **22620** | Job involved shift work | Categorical | 1 Yes  0 No |
| **22630** | Day shifts worked | Categorical | 0 Shift pattern was worked for some of job  1 Shift pattern was worked for whole of job  9 This type of shift pattern was not worked |
| **22631** | Period spent working day shifts | Integer | Duration in job period (years) |
| **22640** | Mixture of day & night shifts | Categorical | 0 Shift pattern was worked for some of job  1 Shift pattern was worked for whole of job  9 This type of shift pattern was not worked |
| **22641** | Period spent working mixed shifts | Integer | Duration in mixed pattern (years) |
| **22642** | Usual night shift length (mixed) | Integer | Hours per night shift |
| **22643** | Night shifts worked monthly (mixed) | Integer | Number of night shifts/month |
| **22644** | Consecutive night shifts (mixed) | Integer | Count of consecutive night shifts |
| **22645** | Rest days during mixed shifts | Integer | Count of rest days within mixed pattern |

Table S15	Sensitivity Analysis: Model 1. Adjusted logistic regression analysis examining the association between every having done shift work and MS diagnosis, controlling for demographic and lifestyle factors in the subset of UK Biobank participants that completed the online work history questionnaire. This analysis (Model 1) accounts for reporting any type of shift work but excludes the variable “Time outdoors” due to potential location on the causal pathway.

|  | **MS Diagnosis** | | |
| --- | --- | --- | --- |
| *Predictors* | *OR* | *CI* | *p* |
| **Ever a Shift Worker (hours)** |  |  |  |
| No (ref) |  |  |  |
| Yes | 0.91 | 0.68 – 1.18 | 0.469 |
| **Early Life Shift Work** |  |  |  |
| No (ref) |  |  |  |
| Yes | 1.33 | 0.94 – 1.89 | 0.108 |
| **Total Work (hours)** | 0.83 | 0.75 – 0.92 | <0.001 |
| **Sex** |  |  |  |
| Female (ref) |  |  |  |
| Male | 0.50 | 0.40 – 0.61 | <0.001 |
| **Age** | 0.98 | 0.97 – 0.99 | <0.001 |
| **Townsend Index** | 0.98 | 0.95 – 1.01 | 0.165 |
| **Childhood Obesity** |  |  |  |
| About average (ref) |  |  |  |
| Plumper | 1.25 | 1.01 – 1.54 | 0.041 |
| Thinner | 0.81 | 0.67 – 0.99 | 0.045 |
| **Alcohol Intake** |  |  |  |
| Never (ref) |  |  |  |
| Special occasions only | 1.01 | 0.69 – 1.49 | 0.963 |
| 1-3 times a month | 0.79 | 0.54 – 1.17 | 0.229 |
| 1-2 times a week | 0.78 | 0.55 – 1.11 | 0.155 |
| 3-4 times a week | 0.60 | 0.42 – 0.87 | 0.006 |
| Daily | 0.74 | 0.52 – 1.08 | 0.108 |
| **Time Outdoors in Summer** |  |  |  |
| **Smoking Status** |  |  |  |
| Never (ref) |  |  |  |
| Previous | 1.20 | 0.99 – 1.44 | 0.060 |
| Current | 2.31 | 1.76 – 3.00 | <0.001 |

Table S16	Sensitivity Analysis: Model 2 Adjusted logistic regression analysis examining the association between total lifetime shift work dose (hours) and MS diagnosis, controlling for demographic and lifestyle factors in the subset of UK Biobank participants that completed the online work history questionnaire. This analysis (Model 2) accounts for reporting any type of shift work but excludes the variable “Time outdoors” due to potential location on the causal pathway.

|  | **MS Diagnosis** | | |
| --- | --- | --- | --- |
| *Predictors* | *OR* | *CI* | *p* |
| **Total Lifetime Shift Work Dose (hr)** | 1.15 | 0.83 – 1.57 | 0.391 |
| **Early Life Shift Work** |  |  |  |
| No (ref) |  |  |  |
| Yes | 1.14 | 0.79 – 1.61 | 0.466 |
| **Total Work (hours)** | 0.82 | 0.74 – 0.91 | <0.001 |
| **Sex** |  |  |  |
| Female (ref) |  |  |  |
| Male | 0.49 | 0.40 – 0.61 | <0.001 |
| **Age** | 0.98 | 0.97 – 0.99 | <0.001 |
| **Townsend Index** | 0.98 | 0.95 – 1.01 | 0.146 |
| **Childhood Obesity** |  |  |  |
| About average (ref) |  |  |  |
| Plumper | 1.25 | 1.01 – 1.54 | 0.041 |
| Thinner | 0.81 | 0.67 – 0.99 | 0.044 |
| **Alcohol Intake** |  |  |  |
| Never (ref) |  |  |  |
| Special occasions only | 1.01 | 0.69 – 1.50 | 0.953 |
| 1-3 times a month | 0.79 | 0.54 – 1.18 | 0.237 |
| 1-2 times a week | 0.78 | 0.56 – 1.12 | 0.168 |
| 3-4 times a week | 0.61 | 0.43 – 0.88 | 0.007 |
| Daily | 0.75 | 0.53 – 1.09 | 0.122 |
| **Shiftwork* Early Life Shift Work** | 0.95 | 0.76 – 1.18 | 0.653 |
| **Smoking Status** |  |  |  |
| Never (ref) |  |  |  |
| Previous | 1.19 | 0.99 – 1.44 | 0.065 |
| Current | 2.29 | 1.74 – 2.97 | <0.001 |

Table S17	Sensitivity Analysis: Model 3 Adjusted logistic regression analysis examining the association between lifetime shift work dose (hours) for each type of shift work (day, night and mix) and MS diagnosis, controlling for demographic and lifestyle factors in the subset of UK Biobank participants that completed the online work history questionnaire. This analysis (Model 3) addresses the specific effects of exposure to day, mixed and night shift work, but excludes the variable “Time outdoors” due to potential location on the causal pathway.

|  | **MS Diagnosis** | | |
| --- | --- | --- | --- |
| *Predictors* | *OR* | *CI* | *p* |
| **Day Shift Work (hours)** | 0.99 | 0.89 – 1.08 | 0.868 |
| **Mixed Shift Work (hours)** | 1.09 | 0.98 – 1.21 | 0.095 |
| **Night Shift Work (hours)** | 0.96 | 0.82 – 1.05 | 0.474 |
| **Early Life Shift Work** |  |  |  |
| No (ref) |  |  |  |
| Yes | 1.11 | 0.80 – 1.51 | 0.534 |
| **Sex** |  |  |  |
| Female (ref) |  |  |  |
| Male | 0.49 | 0.39 – 0.60 | <0.001 |
| **Age** | 0.98 | 0.97 – 0.99 | <0.001 |
| **Townsend Index** | 0.98 | 0.95 – 1.01 | 0.156 |
| **Childhood Obesity** |  |  |  |
| About average (ref) |  |  |  |
| Plumper | 1.25 | 1.01 – 1.54 | 0.041 |
| Thinner | 0.81 | 0.67 – 0.99 | 0.044 |
| **Alcohol Intake** |  |  |  |
| Never (ref) |  |  |  |
| Special occasions only | 1.01 | 0.69 – 1.50 | 0.950 |
| 1-3 times a month | 0.79 | 0.54 – 1.18 | 0.236 |
| 1-2 times a week | 0.78 | 0.56 – 1.12 | 0.165 |
| 3-4 times a week | 0.61 | 0.43 – 0.88 | 0.007 |
| Daily | 0.75 | 0.53 – 1.09 | 0.117 |
| **Smoking Status** |  |  |  |
| Never (ref) |  |  |  |
| Previous | 1.20 | 0.99 – 1.44 | 0.063 |
| Current | 2.30 | 1.75 – 2.99 | <0.001 |

Table S18	Sensitivity Analysis: Adjusted Cox regression analysis of the association between shift work reported at baseline in all working participants of UK Biobank, categorized as either day shift only or any type of night shift and showing the risk of MS diagnosis over 13 years of follow-up. This analysis addresses the specific effects of exposure to day, mixed and night shift work, but excludes the variable “Time outdoors” due to potential location on the causal pathway.

|  | **MS Diagnosis** | | |
| --- | --- | --- | --- |
|  | *HR* | *95 % CI* | *p* |
| **Shift Work Status** |  |  |  |
| No Shift Work (ref) |  |  |  |
| Day Shift Work | 0.86 | 0.56–1.32 | 0.496 |
| Night Shift Work | 1.14 | 0.77–1.68 | 0.519 |
| **Sex** |  |  |  |
| Female (ref) |  |  |  |
| Male | 0.48 | 0.37–0.62 | <0.001 |
| **Age** | 0.95 | 0.94–0.97 | <0.001 |
| **Townsend Index** | 0.99 | 0.95–1.03 | 0.472 |
| **Childhood Obesity** |  |  |  |
| About average (ref) |  |  |  |
| Plumper | 1.00 | 0.74–1.35 | 0.993 |
| Thinner | 0.77 | 0.59–1.00 | 0.053 |
| **Chronotype** |  |  |  |
| Morning (ref) |  |  |  |
| More morning than evening | 0.82 | 0.55–1.21 | 0.313 |
| More evening than morning | 0.93 | 0.63–1.36 | 0.708 |
| Evening | 0.82 | 0.55–1.21 | 0.313 |
| **Alcohol Intake** |  |  |  |
| Never (ref) |  |  |  |
| Special occasions only | 0.78 | 0.47–1.29 | 0.337 |
| 1-3 times a month | 0.72 | 0.44–1.20 | 0.210 |
| 1-2 times a week | 0.76 | 0.48–1.20 | 0.226 |
| 3-4 times a week | 0.69 | 0.43–1.10 | 0.119 |
| Daily | 0.69 | 0.42–1.14 | 0.137 |
| **Smoking Status** |  |  |  |
| Never (ref) |  |  |  |
| Previous | 1.06 | 0.81–1.39 | 0.665 |
| Current | 2.06 | 1.52–2.79 | <0.001 |

Table S19	Details of the numbers and percentages of the total number of participants that reported shift work in the work history questionnaire.

|  | **Control (N=119,665)** | **Multiple Sclerosis (N=574)** | **p** |
| --- | --- | --- | --- |
| **Early Life Night Shift Worker** | 9,491 (8 %) | 53 (9 %) | 0.283 |
| **Early Life Shift Worker** | 12,095 (10 %) | 65 (11 %) | 0.371 |
| **Ever a Shift Worker** | 26,641 (22 %) | 127 (22 %) | 0.877 |
| **Ever a Night Shift Worker** | 20,410 (17 %) | 96 (17 %) | 0.977 |
|  | |  |  |

Table S20	Details of the numbers and percentages of the total number of participants that reported shift work at baseline

|  | **Control (n=287,957)** | **MS Diagnosis (n=342)** | **p** |
| --- | --- | --- | --- |
| **Current Shift Worker** |  |  | 0.175 |
| Always | 22,024 (8 %) | 31 (9 %) |  |
| Never/rarely | 235,704 (82 %) | 277 (81 %) |  |
| Sometimes | 21,167 (7 %) | 18 (5 %) |  |
| Usually | 6,071 (2 %) | 11 (3 %) |  |
| Missing | 2991 (1.0%) | 5 (1.5%) |  |
| **Current Night Shift Worker** |  |  | 0.232 |
| Always | 7,101 (2 %) | 12 (4 %) |  |
| Never/rarely | 24,400 (8 %) | 27 (8 %) |  |
| Sometimes | 14,200 (5 %) | 14 (4 %) |  |
| Usually | 3,924 (1 %) | 11 (3 %) |  |
| Missing | 2991 (1.0%) | 5 (1.5%) |  |

Table S21 Adjusted logistic regression analysis examining the association between lifetime dose of day, night and mixed Shift Work) and MS diagnosis, controlling for demographic and lifestyle factors in the subset of UK Biobank participants that completed the online work history questionnaire. This analysis (Model 3) addresses the specific effects of exposure to day, mixed and night shift work. Note that OR are standardised and should be interpreted as change per unit standard deviation increase.

|  | **MS Diagnosis** | | |
| --- | --- | --- | --- |
| *Predictors* | *OR* | *CI* | *p* |
| **Day Shift Work (hours)** | **1.00** | 0.90 – 1.10 | 0.930* |
| **Mixed Shift Work (hours)** | **1.12** | 1.01 - 1.24 | 0.094* |
| **Night Shift Work (hours)** | **0.94** | 0.83 – 1.06 | 0.892* |
| **Early Life Shift Work** |  |  |  |
| No (ref) |  |  |  |
| Yes | 1.05 | 0.75 – 1.45 | 0.766 |
| **Total Work (hours)** | 0.81 | 0.73 – 0.91 | **<0.001** |
| **Sex** |  |  |  |
| Female (ref) |  |  |  |
| Male | 0.50 | 0.40 – 0.62 | **<0.001** |
| **Age** | 0.98 | 0.97 – 0.99 | **0.002** |
| **Townsend Index** | 0.98 | 0.95 – 1.01 | 0.131 |
| **Childhood Obesity** |  |  |  |
| About average (ref) |  |  |  |
| Plumper | 1.17 | 0.94 – 1.46 | 0.151 |
| Thinner | 0.81 | 0.66 – 0.99 | **0.038** |
| **Alcohol Intake** |  |  |  |
| Never (ref) |  |  |  |
| Special occasions only | 0.99 | 0.67 – 1.47 | 0.951 |
| 1-3 times a month | 0.79 | 0.54 – 1.18 | 0.244 |
| 1-2 times a week | 0.75 | 0.53 – 1.09 | 0.121 |
| 3-4 times a week | 0.59 | 0.41 – 0.86 | **0.005** |
| Daily | 0.71 | 0.50 – 1.04 | 0.071 |
| **Time Outdoors in Summer** | 0.90 | 0.86 – 0.95 | **<0.001** |
| **Smoking Status** |  |  |  |
| Never (ref) |  |  |  |
| Previous | 1.22 | 1.00 – 1.47 | **0.045** |
| Current | 2.43 | 1.84 – 3.16 | **<0.001** |
|  |  |  |  |

*p-values were adjusted for multiple comparisons using the Benjamini-Hochberg procedure

Figure S1	Screenshot of the touchscreen questions used to collect shift work data at baseline.

Figure S2	Screenshot of the online questions used to collect lifetime shift work data in the online follow up questionnaire.

Figure S3	Directed acyclic graph (DAG) of shift work and multiple sclerosis (MS) diagnosis. Demographic and lifestyle factors were included as potential confounders, while sleep duration and daylight exposure are shown as possible mediators of the association between shift work and MS. Sensitivity analysis did not justify their role as mediators, and they are included as covariables and potential confounders in the models in this study, although their potential mediating effects are not excluded.

**Shift Work**

**MS Diagnosis**

Age, Sex, Ethnicity SEP

Smoking Alcohol Exercise Obesity

Daylight Exposure

Circadian & Sleep Disruption

*Mediator?*

*Confounder*

**DIRECT**

*Confounder*

*Mediator?*
